# Dynamic Margins of Stability During Robot-Assisted Walking in Able-Bodied Individuals: A Preliminary Study

**DOI:** 10.3389/frobt.2020.574365

**Published:** 2020-12-09

**Authors:** Arvind Ramanujam, Kamyar Momeni, Manikandan Ravi, Jonathan Augustine, Erica Garbarini, Peter Barrance, Ann M. Spungen, Pierre Asselin, Steven Knezevic, Gail F. Forrest

**Affiliations:** ^1^Kessler Foundation, West Orange, NJ, United States; ^2^Koneksa Health, New York, NY, United States; ^3^Rutgers, New Jersey Medical School, Newark, NJ, United States; ^4^James J. Peters Veterans Affairs Medical Center, Bronx, NY, United States

**Keywords:** robotic exoskeleton, stability, kinematics, gait, center of mass

## Abstract

**Background:** Gait analysis studies during robot-assisted walking have been predominantly focused on lower limb biomechanics. During robot-assisted walking, the users' interaction with the robot and their adaptations translate into altered gait mechanics. Hence, robust and objective metrics for quantifying walking performance during robot-assisted gait are especially relevant as it relates to dynamic stability. In this study, we assessed bi-planar dynamic stability margins for healthy adults during robot-assisted walking using EksoGT™, ReWalk™, and Indego® compared to independent overground walking at slow, self-selected, and fast speeds. Further, we examined the use of forearm crutches and its influence on dynamic gait stability margins.

**Methods:** Kinematic data were collected at 60 Hz under several walking conditions with and without the robotic exoskeleton for six healthy controls. Outcome measures included (i) whole-body center of mass (CoM) and extrapolated CoM (X_CoM_), (ii) base of support (BoS), (iii) margin of stability (MoS) with respect to both feet and bilateral crutches.

**Results:** Stability outcomes during exoskeleton-assisted walking at self-selected, comfortable walking speeds were significantly (*p* < 0.05) different compared to overground walking at self-selected speeds. Unlike overground walking, the control mechanisms for stability using these exoskeletons were not related to walking speed. MoSs were lower during the single support phase of gait, especially in the medial–lateral direction for all devices. MoSs relative to feet were significantly (*p* < 0.05) lower than those relative to crutches. The spatial location of crutches during exoskeleton-assisted walking pushed the whole-body CoM, during single support, beyond the lateral boundary of the lead foot, increasing the risk for falls if crutch slippage were to occur.

**Conclusion:** Careful consideration of crutch placement is critical to ensuring that the margins of stability are always within the limits of the BoS to control stability and decrease fall risk.

## Introduction

Commercially available exoskeletons, such as the EksoGT™ (Ekso Bionics, Richmond, CA), ReWalk™ (ReWalk Robotics, Inc., Marlborough, MA), and Indego® (Parker Hannifin Corp, Cleveland, OH), are suggested rehabilitative modalities for overground (OG) walking among individuals with movement limitations (U.S. Food Drug Administration, 2014, 2016a,b, 2017). Walking using these exoskeletons requires assistive devices like bilateral canes, forearm crutches, or a walker; however, these assistive devices can inhibit dynamic stability (Bateni and Maki, 2005; Saunders et al., 2013). Additionally, slipping or sliding of bilateral cane and crutch tips due to the material used or different walking surfaces (e.g., wet pavements, snow, ice) can lead to further injuries (Kennaway, 1970; Bennett and Murphy, 1977). Therefore, understanding the posture and balance control strategies during robotic exoskeleton (RE) gait compared to independent OG walking is crucial in ensuring the safety of these individuals and preventing falls. Although researchers have studied the kinematic, spatiotemporal, cardio-pulmonary, cognitive, neuromuscular, and safety outcomes associated with RE training (Nozaki et al., 2005; Sayenko et al., 2015; Miller et al., 2016; Ramanujam et al., 2017, 2018, 2019a; Saleh et al., 2017; Gordon et al., 2018; Tefertiller et al., 2018; Forrest et al., 2019; Guanziroli et al., 2019; Khan et al., 2019; Luger et al., 2019; Momeni et al., 2019; Wang et al., 2019; Yildirim et al., 2019; McIntosh et al., 2020), a thorough assessment of dynamic stability during RE walking is important to understanding the mechanics of human–machine interactions during exoskeleton-assisted gait and the potential to lower fall risk.

Research studies involving gait analysis during RE-assisted gait have been predominantly focused on lower limb biomechanics (Sylos-Labini et al., 2014; Louie et al., 2015; Ramanujam et al., 2017, 2018, 2019a,b; Husain et al., 2018; Forrest et al., 2019). With the advances in research and development of powered lower limb exoskeletons (Jiménez-Fabián and Verlinden, 2012; Molteni et al., 2018), optimal exoskeleton choice depends on a variety of factors including the design and control of the device, user's ability, task, and environment. During RE-assisted walking, the users' interaction with the RE and their adaptation to the subtle differences between the devices translate into altered gait mechanics (Ramanujam et al., 2018, 2019a). As a result, robust and objective metrics for quantifying walking performance during RE-assisted gait are especially relevant as it relates to dynamic stability.

Whole-body center of mass (CoM) is a key determinant for balance control mechanisms in the quantification of dynamic gait stability (Kaya et al., 1998; Lee and Chou, 2006). Several authors have used CoM to describe postural sway, symmetry, and stability (Kaya et al., 1998; Lee and Chou, 2006; McAndrew Young et al., 2012; Ramanujam et al., 2019a,b). The instantaneous position and velocity of the whole-body CoM in relation to the base of support (BoS) has been used previously to calculate margins of stability (MoSs) (Hof et al., 2005) and evaluate the step-to-step changes in MoS during walking.

Our group has recently published articles to assess the posture and balance of individuals with spinal cord injury (SCI) and able-bodied (AB) controls during RE-assisted gait with forearm crutches in the EksoGT™ and ReWalk™ by examining their instantaneous three-dimensional CoM excursions (whole body, trunk, and lower extremity) with respect to the BoS (Ramanujam et al., 2019a,b). As an extension to our already-published work, in this study, we used the instantaneous CoM measures to further compute the dynamic stability margins for healthy adults during RE walking using EksoGT™, ReWalk™, and Indego® at self-selected, comfortable, and safe walking speeds to test our hypothesis that the MoS measures during RE-assisted walking will differ based on the device and assist mode, compared to independent OG walking at a self-selected speed. Additionally, we assessed fast and slow walking speeds during OG walking to evaluate the effect of speed on stability outcomes. Further, we examined the control of stability during the different phases of a gait cycle and the influence of forearm crutches in conjunction with RE walking on dynamic gait stability margins.

## Methods

### Participant Demographics

Six male AB individuals (age: 29.50 ± 4.97 years, weight: 82.57 ± 13.23 kg, height: 1.80 ± 0.07 m) completed an informed consent form, approved by the Kessler Foundation and James J. Peters Veterans Affairs Medical Center Institutional Review Boards, to participate in the study. The inclusion and exclusion criteria have been previously reported (Ramanujam et al., 2019a).

### Data Collection

AB individuals were first trained, under the guidance of a physical therapist, to walk independently with each RE under minimal supervision using bilateral forearm crutches before the scheduled data collection session. Individuals were asked to walk across a 10 m walkway multiple times under several walking conditions (Table 1) with and without the RE as previously reported (Ramanujam et al., 2019a). Kinematic data (Motion Analysis Corporation, Santa Rosa, CA) were collected at 60 Hz, and data from at least 10 gait cycles per condition were used for further analysis.

**Table 1A:** Demographics and testing conditions.

	**AB**	**AB**	**AB**	**AB**	**AB**	**AB**
	**1**	**2**	**3**	**4**	**5**	**6**
**Age (years)**	38	30	26	26	25	32
**Weight (Kg)**	86.4	104.5	81.8	84.1	65.9	72.7
**Height (m)**	1.83	1.78	1.90	1.80	1.70	1.80

**Table 1B:** Walking conditions.

**1**	Walking with Indego^®^ exoskeleton
**2**	Walking with ReWalk™ exoskeleton
**3**	Walking with EksoGT™ exoskeleton in *2Free* mode
**4**	Walking with EksoGT™ exoskeleton in *Fixed-Assist* mode
**5**	Walking with EksoGT™ exoskeleton in *Max-Assist* mode
**6**	Overground walking at FAST speed
**7**	Overground walking at self-selected (SS) speed
**8**	Overground walking at SLOW speed

**Table 1C:** List of abbreviations.

**SCI**	-	Spinal Cord Injury
**RE**	-	Robotic Exoskeleton
**CoM**	-	Center of Mass
**X_CoM_**	-	Extrapolated CoM
**BoS**	-	Base of Support
**MoS**	-	Margin of Stability
**OG**	-	Overground
**AP**	-	Anterior-posterior
**ML**	-	Medial-lateral
**AB**	-	Able-Bodied
**SS**	-	Self-Selected
**F**	-	Foot
**C**	-	Crutch
**RMSD**	-	Root Mean Squared Difference
**IDS**	-	Initial Double Support
**SLS**	-	Single Limb Support
**TDS**	-	Terminal Double Support
**SW**	-	Swing

### Exoskeleton Settings and Training Modes

Individuals were fitted with the RE devices per anthropometric measurements and by adjusting segments of the exoskeleton. The settings and operating principle for all RE devices tested are listed in Table 2 (EksoGT™ Operating Manual Copyright 2013 Ekso GT Bionics, Inc Part Number 103299 REV B1). OG walking conditions, without the RE, included walking at FAST, self-selected (SS), and SLOW speeds. Individuals were given a few practice walks at these three speeds (SLOW, SS, and FAST) and instructed to walk at their own safe and comfortable pace before kinematic data were collected. All walking trials with the REs were collected with the use of forearm crutches at their self-selected, comfortable, and safe walking speeds. The RE training modes (Table 2), with the addition of Indego® as an additional device, were selected based on our previous work (Ramanujam et al., 2019b) on individuals with SCI and AB controls and on the individual's ability to walk independently with minimal supervision.

**Table 2:** Robotic exoskeleton operating principle and settings.

	**Device operating principle**	**Device settings**
**EksoGT**™	Lateral weight shift onto one foto complete the stepping on the contralateral foot combined with moving the contralateral crutch forward	*Max-Assist* (100%): *Max-Assist* provides the maximum amount of motor assistance to the user at all times where the leg moves consistently through swing and is less susceptible to the user's interaction. *Fixed-Assist* (100–0%): *Fixed-Assist* is when the forward motor assistance is set to a fixed maximum value provided throughout the swing phase. In this study, the Fixed-Assist modes chosen were 0, 35, and 60%. *2Free*: *2Free* mode signals that the leg is not being controlled by motors and programming and is free to move under the user's control.
**ReWalk**™	Initiates a step by tilting the trunk anteriorly and moving both crutches forward simultaneously	Hip angle = 25°, knee angle = 37°, velocity/swing time = 600 ms, tilt = 7°
**Indego**^®^	Postural cues with predefined step trajectory to trigger all transitions (e.g., to walk forward, the user just leans forward)	Motion+ mode (assist = 20%, speed = medium, length = long, step height = medium)

Training and testing with the EksoGT™ was performed under multiple conditions and swing assist modes, which provide adaptive assistance during the swing phase of the gait. These modes include “Max-Assist,” “Fixed-Assist,” and “2Free.” The Max-Assist mode provides a constant, maximum amount of motor power (100%) to move a user's leg through the trajectory-controlled swing phase. The Fixed-Assist mode provides assistance throughout the trajectory-controlled swing phase up to a predetermined value that is set as a percentage of the maximum amount of motor power (100–0%). In other words, if users complete the swing phase with their own strength, without using the power of the motors, the value set on the Fixed-Assist mode would make no difference in the process. For instance, at 35% Fixed-Assist, the individual may use up to, or less than, 35% of the maximum motor power to complete the swing phase with a predetermined trajectory. Similarly, 0% Fixed-Assist requires individuals to utilize their own effort to finish the swing phase without using any amount of motor power; if they fail to do so, a safety feature will initiate to complete the swing phase. The 2Free mode allows users to freely move their leg with their own strength without being constrained to a predetermined swing trajectory.

In this study, to cover the entire range of available Fixed-Assist levels (100–0%) and also match the assist levels used while training individuals with SCI (depending on their ability and the therapist's recommendations) (Ramanujam et al., 2019b), we chose 0, 35, and 60% as the levels at which the device was tested for every individual. In Fixed-Assist mode, the goal is to encourage the participant to provide maximal effort in order to complete the swing phase while receiving up to a ceiling amount of motor power in assistance. We collected three to five trials (at least 10 complete gait cycles) at 0, 35, and 60% fixed assistance levels as a low, moderate, and high level of assistance, respectively, and calculated average profiles across these conditions for the Fixed-Assist mode.

### Data Analysis

**Figure 1:**
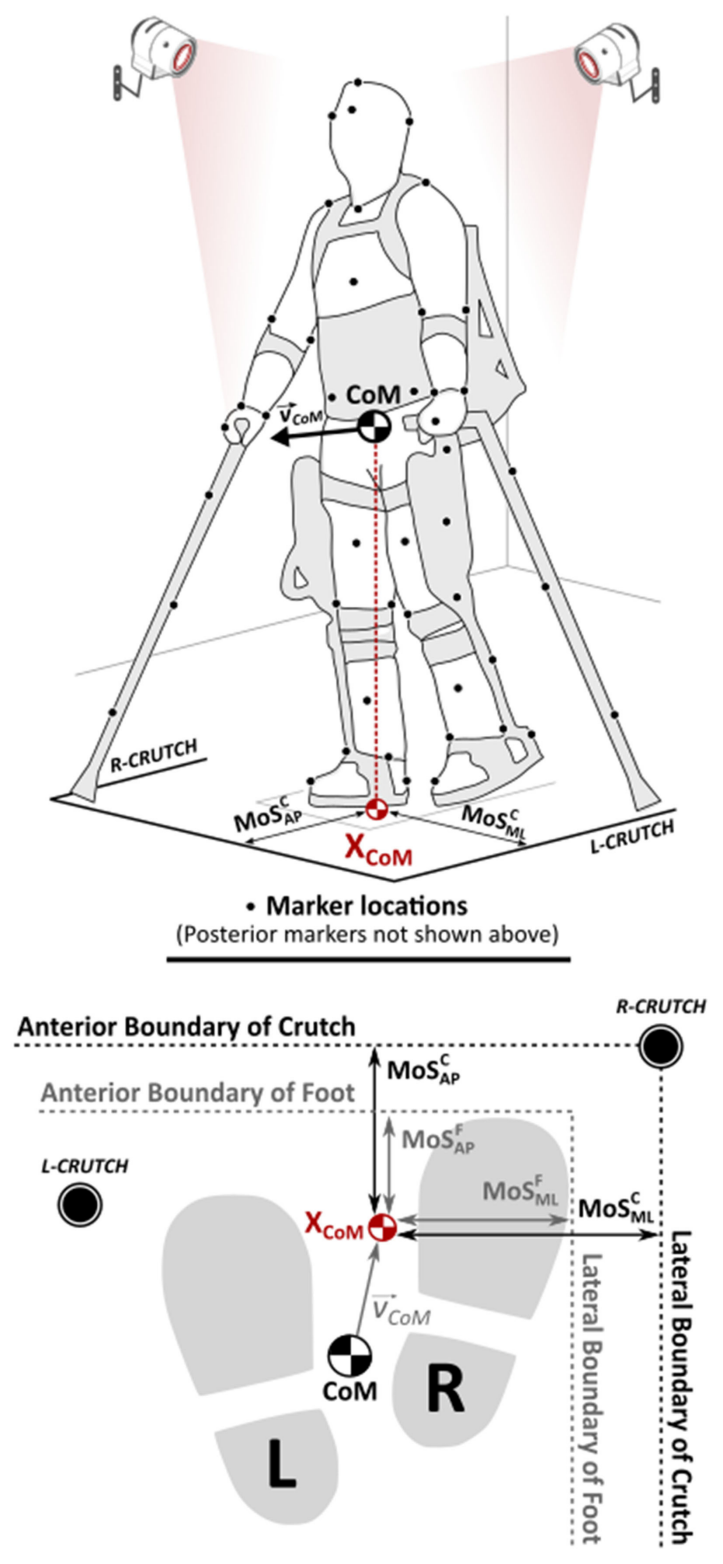
Center of mass (CoM) representation and definitions for the margins of stability (MoSs) with respect to the feet and bilateral crutches.

Kinematic data were filtered, time-normalized, and averaged across multiple gait cycles to create mean kinematic profiles (Ramanujam et al., 2019a). The outcome measures calculated from kinematics using custom written programs in MATLAB (MathWorks®, Natick, MA) include: (i) whole-body CoM and the velocity-adjusted extrapolated CoM (X_CoM_), (ii) margin of stability (MoS) in the anterior–posterior (AP) and medial–lateral (ML) directions with respect to both feet (MoS^F^) and bilateral crutches (MoS^C^), and (iii) walking speed; see Figure 1.

MoS parameters (Figure 1) were computed using the instantaneous CoM and X_CoM_ values (Hof, 2008). The coefficient of correlation (*R*) was calculated to examine relationships between MoS outcomes and walking speed and categorized into weak (|*R*| ≤ 0.40), moderate (0.40 < |*R*| ≤ 0.60), strong (0.60 < |*R*| ≤ 0.80), and very strong (0.80 < |*R*| ≤ 1) correlations (Evans, 1996). First-return plots (McAndrew Young et al., 2012) to assess stride-to-stride variability in MoS outcomes were generated (MoS_i_ vs. MoS_i−1_), and root mean squared differences (RMSD) were computed to compare the variance from the 45° line of symmetry. MoS outcomes were computed at the point of heel-strike (bilaterally). Also, the least stable point (lowest MoS value and its associated T) during each phase of a gait cycle (i.e., initial double support, IDS: ipsilateral foot strike to contralateral foot off; single limb support, SLS: contralateral foot off to contralateral foot strike; terminal double support, TDS: contralateral foot strike to ipsilateral foot off; and swing, SW: ipsilateral foot off to subsequent ipsilateral foot strike) was identified and represented as a stability matrix.

### Margins of Stability

As shown in Figure 1, the instantaneous location of the whole-body CoM and the X_CoM_ were calculated from kinematics.

(1) $$\begin{array}{l} X_{CoM}=c+\dot{c}/\omega_{0} \end{array}$$

(2) $$\begin{array}{l} \omega_{0}=\sqrt{g/l} \end{array}$$

The CoM position and velocity components are denoted as c and *ċ* respectively, “g” = 9.81 m/s^2^ (gravitational constant) with an oscillation frequency of “0,” and “l” (equivalent pendulum length) was the mean distance from the heel marker to the CoM at heel-strike. The X_CoM_ was then projected onto the floor (transverse plane) to establish its relationship with respect to the boundaries of BoS, bilaterally. BoS was defined as the linear distance between the boundaries (AP and ML) of the farthest support points in contact with the ground. In this study, BoS was calculated with respect to bilateral feet as well as crutches. The lateral boundary of BoS (left or right, foot or crutch) for MoS calculations was chosen so as to match the directionality of CoM velocity.

Different combinations of dynamic MoS were then calculated in the AP and ML directions with respect to (i) BoS between the feet (MoS^F^) and (ii) BoS defined by the bilateral crutches (MoS^C^) as follows:

(3) $$\begin{array}{l} {MoS}_{j}^{i}=X_{{CoM}_{j}}-{BoS}_{j}^{i} \end{array}$$

where

(4) $$\begin{array}{l} i=F\;or\;C \end{array}$$

(5) $$\begin{array}{l} j=AP\;or\;ML \end{array}$$

A higher positive value for MoS is associated with greater stability. Negative MoS values are considered unstable. Further, the MoS values were normalized to CoM velocity to calculate a temporal index (T) indicative of stability as defined below.

(6) $$\begin{array}{l} T_{j}^{i}\;=\;{MoS}_{j}^{i}/{\dot{c}}_{j} \end{array}$$

T values represent the temporal deviation (in seconds) from the limits of BoS. Similar to the sign convention for MoS, a positive T value indicates the time available until the point of instability, while T < 0 is indicative of the time elapsed beyond the point of instability. Hence, higher positive or negative T values represent greater stability or instability, respectively.

### Statistical Analysis

A multivariate analysis of variance (ANOVA), to compare RE and OG walking conditions, was performed for all outcome variables using Bonferroni correction. Tukey *post hoc* tested for all possible two-way comparisons. Paired-sample *t*-tests were used to compare the means across devices and modes, as well as OG walking conditions (*p* < 0.05).

## Results

### MoS Outcomes During OG vs. RE Walking

**Figure 2:**
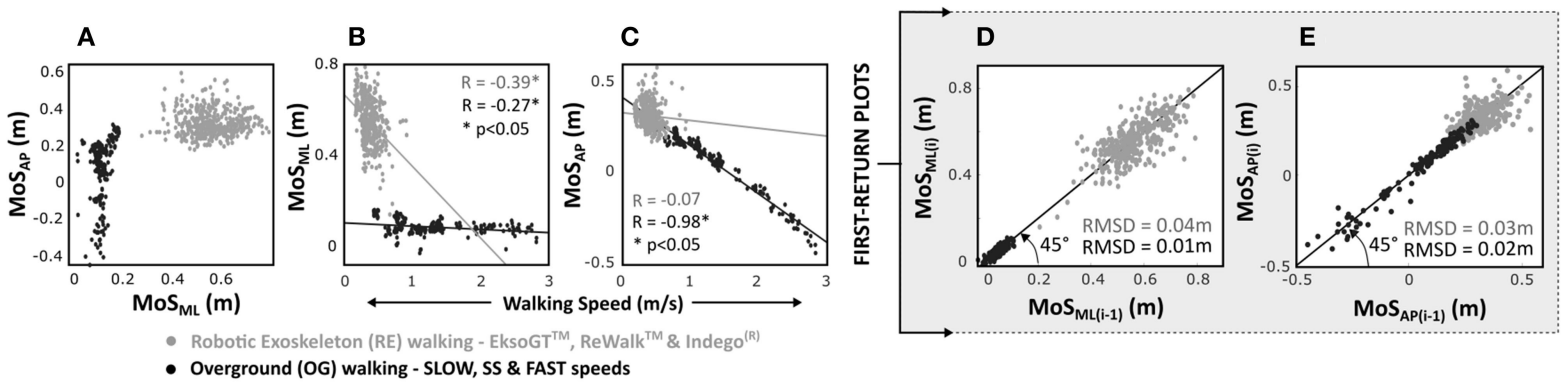
MoS outcomes in anterior–posterior (AP) and medial–lateral (ML) directions grouped by overground (OG) and robotic exoskeleton (RE) walking for able-bodied individuals at heel-strike. Correlations for **(A)** MoS_AP_ vs. MoS_ML_, **(B)** MoS_ML_, and **(C)** MoS_AP_ vs. walking speed and first-return plots for **(D)** MoS_ML_ and **(E)** MoS_AP_.

At heel-strike during RE walking across all tested devices and conditions, the overall mean MoSs calculated using the BoS defined by the bilateral crutches (MoS_ML_ = 0.56 ± 0.09 m; MoS_AP_ = 0.31 ± 0.08 m) were significantly (*p* < 0.05) greater than OG walking (MoS_ML_ = 0.11 ± 0.03 m; MoS_AP_ = 0.06 ± 0.17 m) across all speeds in both directions (Figure 2A).

For RE walking, a weak negative correlation was observed for MoS_ML_ (*R* = −0.39) vs. walking speed (*R* = −0.07 for MoS_AP_). During OG walking, the correlation between MoS_ML_ and walking speed was weak (*R* = −0.27); however, the correlation for MoS_AP_ was significantly (*p* < 0.05) strong and negative (*R* = −0.98, Figures 2B,C).

First-return plots (Figures 2D,E) showed that overall RMSD values were significantly (*p* < 0.05) greater for RE walking compared to OG walking in both AP (RMSD_RE−AP_ = 0.03 ± 0.02 m; RMSD_OG−AP_ = 0.02 ± 0.02 m) and ML (RMSD_RE−ML_ = 0.04 ± 0.03 m; RMSD_OG−ML_ = 0.01 ± 0.01 m) directions.

### Effect of Device Settings and Speed on MoS

**Figure 3:**
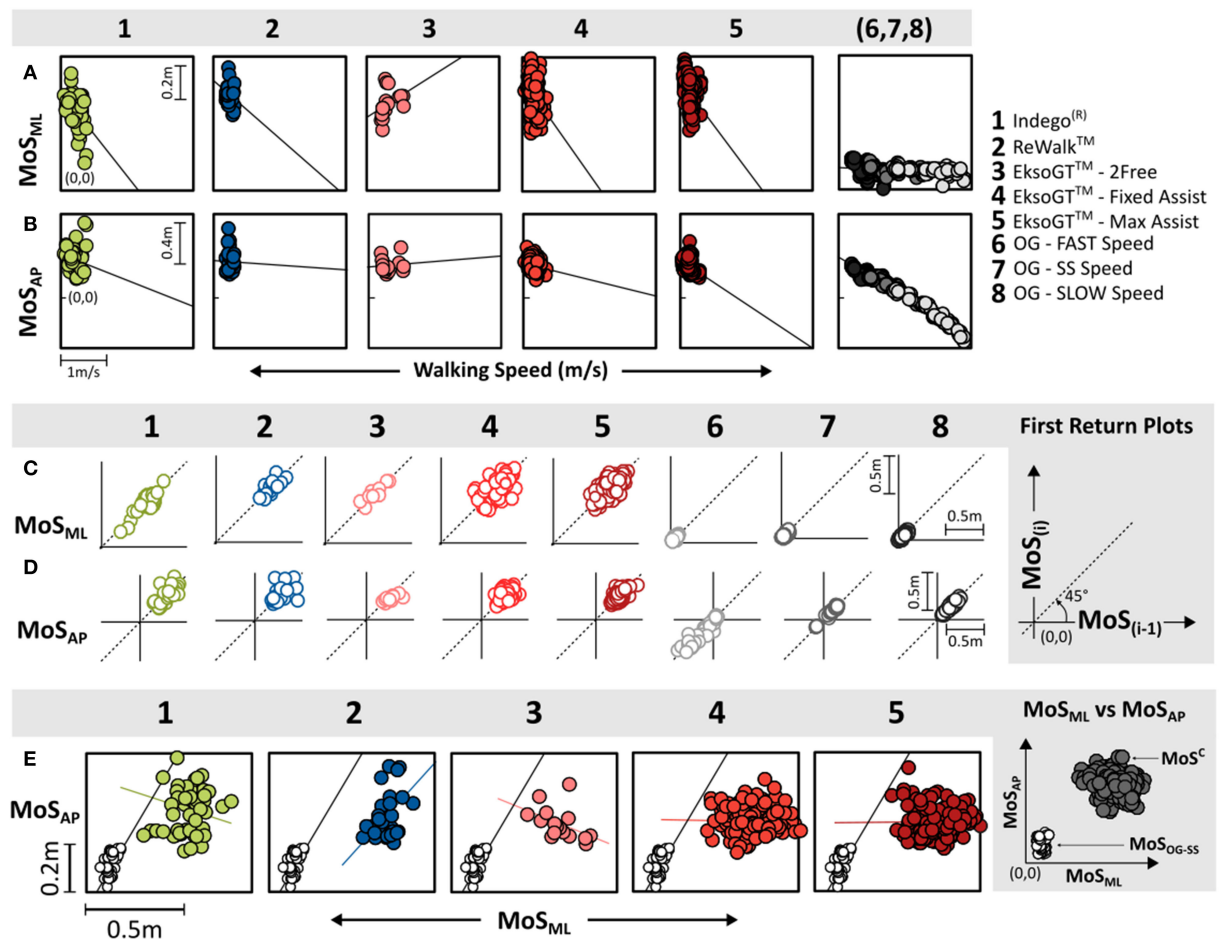
MoS outcomes per condition: correlations for walking speed with MoS outcomes in the **(A)** ML and **(B)** AP directions. Stride-to-stride variability shown using first-return plots for MoS in the ML **(C)** and AP **(D)** directions. **(E)** MoS_AP_ vs. MoS_ML_ correlations for OG walking at self-selected (SS) speed and RE walking. MoS outcomes shown for conditions 1–5 are with respect to bilateral crutches, while OG walking conditions (6–8) occurred without crutches.

A significant moderate negative correlation (*R* = −0.48, *p* < 0.05) was observed for MoS_ML_ with walking speed for the Indego®. For EksoGT™ in the *Max-Assist* mode, the correlation was weak (*R* = −0.37) for MoS_ML_ with speed, while it was moderate (*R* = −0.42, *p* < 0.05) for MoS_AP_ (Figures 3A,B and Table 3). In the *Fixed-Assist* mode, the correlations were relatively weaker (|*R*| < 0.36) and significantly negative (*p* < 0.05) in both directions during EksoGT™ walking. For OG walking, the correlations for MoS_ML_ and walking speeds were moderately negative (−0.51 < *R* < −0.42, p<0.05); however, they were significantly very strong for MoS_AP_ (−0.98 < *R* < −0.89, *p* < 0.05).

**Table 3:** Margin of stability (MoS) outcomes across robotic exoskeleton (RE) devices and overground (OG) walk conditions at heel-strike.

				**RE walking conditions**					OG walking conditions^**^		
				**1**	**2**	**3**	**4**	**5**	**6**	**7**	**8**
${\text{MoS}}_{\text{ML}}^{\text{F}}$			*(m)*	0.11 ± *0.02*	0.18 ± *0.01*	0.12 ± *0.02*	0.13 ± *0.02*	0.13 ± *0.03*	0.11 ± *0.02*	0.11 ± *0.02*	0.11 ± *0.04*
${\text{MoS}}_{\text{ML}}^{\text{C}}$			*(m)*	0.48 ± *0.09*	0.57 ± *0.06*	0.52 ± *0.09*	0.58 ± *0.09*	0.58 ± *0.09*	*- Walking without crutch -*		
${\text{MoS}}_{\text{AP}}^{\text{F}}$			*(m)*	0.22 ± *0.03*	0.24 ± *0.02*	0.23 ± *0.03*	0.26 ± *0.04*	0.26 ± *0.03*	−0.16 ± *0.15*	0.09 ± *0.08*	0.18 ± *0.06*
${\text{MoS}}_{\text{AP}}^{\text{C}}$			*(m)*	0.36 ± *0.12*	0.33 ± *0.11*	0.29 ± *0.07*	0.30 ± *0.05*	0.30 ± *0.06*	*- Walking without crutch -*		
${\text{MoS}}_{\text{ML}}^{\text{C}}$	vs.	Walking speed	*R*	−0.48^*^	−0.23	0.36	−0.36^*^	−0.37^*^	−0.51^*^	−0.42^*^	−0.49^*^
${\text{MoS}}_{\text{AP}}^{\text{C}}$	vs.	Walking speed	*R*	−0.20	−0.02	0.09	−0.18^*^	−0.42^*^	−0.98^*^	−0.96^*^	−0.89^*^
${\text{MoS}}_{\text{AP}}^{\text{C}}$	vs.	${\text{MoS}}_{\text{ML}}^{\text{C}}$	*R*	−0.24	0.52^*^	−0.45	−0.03	0.01	0.48^*^	0.40^*^	0.59^*^
${\text{MoS}}_{\text{ML(i)}}^{\text{C}}$	vs.	${\text{MoS}}_{\text{ML(i - 1)}}^{\text{C}}$	*RMSD (m)*	0.03 ± *0.02*	0.03 ± *0.02*	0.02 ± *0.02*	0.04 ± *0.03*	0.04 ± *0.03*	0.01 ± *0.01*	0.01 ± *0.01*	0.01 ± *0.01*
${\text{MoS}}_{\text{AP(i)}}^{\text{C}}$	vs.	${\text{MoS}}_{\text{AP(i - 1)}}^{\text{C}}$	*RMSD (m)*	0.04 ± *0.05*	0.06 ± *0.06*	0.04 ± *0.04*	0.03 ± *0.03*	0.02 ± *0.02*	0.03 ± *0.03*	0.01 ± *0.01*	0.01 ± *0.01*
Walking Speed			*(m/s)*	0.47 ± *0.13*	0.48 ± *0.06*	0.61 ± *0.17*	0.37 ± *0.08*	0.31 ± *0.09*	2.10 ± *0.49*	1.24 ± *0.32*	0.84 ± *0.26*

*Walking conditions 1 to 8 are as described previously*;

** *OG walking occurred without the use of crutches; values are presented as mean ± SD or number*;

* *p < 0.05*.

First-return plots (Figures 3C,D) showed that RMSD values across AP and ML directions were the lowest for OG walking across all speeds compared to all RE walking and settings (Table 3). For OG walking, faster speeds produced greater RMSD_AP_ values. Walking in the EksoGT™ under the *Max-Assist* and *Fixed-Assist* modes was associated with greater RMSD values in the ML direction, while walking in the EksoGT™ (*2Free* mode), Indego®, and ReWalk™ produced greater RMSD values in the AP direction (Figures 3C,D and Table 3). In both directions, RMSD values for the RE devices were significantly (*p* < 0.05) greater than OG walking, except for EksoGT™ (*2Free* mode). Correlations between MoS_ML_ and MoS_AP_ (Figure 3E) were moderate (0.40 < *R* < 0.59) and significantly positive (*p* < 0.05) for OG walking across all speeds. For RE walking, only ReWalk™ produced positive correlations for these measures (*R* = 0.52, *p* < 0.05).

### MoS Representation Referenced to Feet vs. Crutches

MoS outcomes calculated relative to bilateral foot were significantly lower (MoS^F^ < 0.13 m, *p* < 0.05) compared to those relative to bilateral crutches (MoS^C^ > 0.48 m). MoS^F^ values for Indego® (0.11 m) and EksoGT™ (*2Free* mode, 0.12 m) were not significantly different from OG walking in the ML direction, while it was the highest for ReWalk™ (0.18 m, *p* < 0.05). In the AP direction, although less than MoS^C^ (>0.29 m), MoS^F^ values (<0.26 m) were still significantly (*p* < 0.05) greater than OG walking at all speeds. For Indego®, MoS_AP_ outcomes when referenced to bilateral crutches (MoS^C^) were the highest amongst all RE devices; however, it was the lowest when referenced to the feet (MoS^F^).

### Stability Matrix

**Figure 4:**
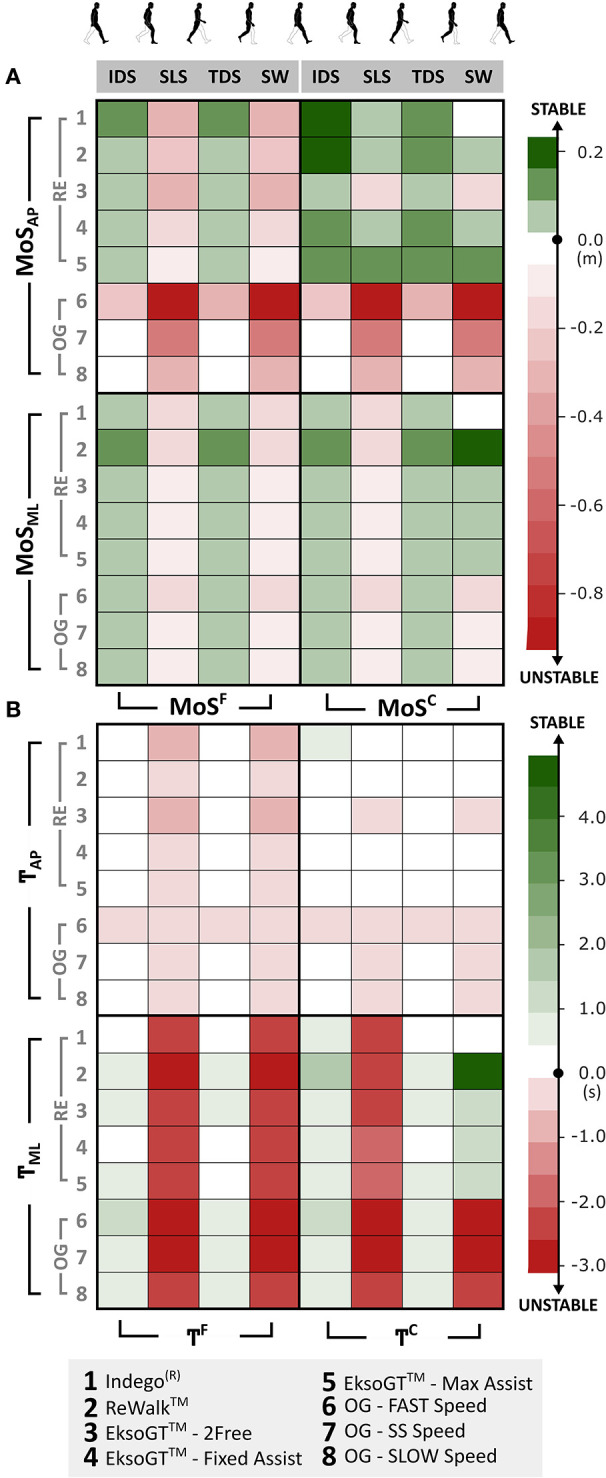
**(A)** Stability matrix showing the lowest stability values (MoS_min_) in both AP and ML directions during each phase of gait cycle [initial double support (IDS), single support (SS), terminal double support (TDS), and SWING] across RE and OG walking. **(B)** MoS normalized to CoM velocity (referred to as “temporal stability”—?) within each phase of the gait cycle.

In addition to MoS outcomes computed at the point of heel-strike, the least stable point (MoS_min_) during each phase of a gait cycle (IDS, SLS, TDS, and SW) was also determined (Figure 4). Medial–laterally, MoS^C^ and MoS^F^ stayed positive (0.05 m < MoS_ML_ < 0.11 m) during IDS and TDS. With RE walking, MoS_ML_ was highest with the ReWalk™ during IDS and TDS (> 0.10 m). Conversely, MoS values were negative during SS and SW. In the AP direction, during RE walking, MoS^C^ was positive during the entire gait cycle except for EksoGT™ in the *2Free* mode that experienced negative values (<-0.16 m) during SS and SW, while MoS^F^ was found to be negative (<-0.11 m) during the SS and SW. Between OG and RE walking, MoS_AP_ values were consistently lower during OG walking across all speeds (especially faster speeds) and gait phases (especially SS).

Figure 4B shows the velocity-normalized representation of MoS (referred to as “T,” temporal stability), which was calculated by dividing the MoS values in Figure 4A by the mean directional CoM velocity during the corresponding phase of the gait cycle (see Equation 4). During OG and RE walking, bidirectional T^F^ was negative (−3.11 s < T^F^ < −0.39 s) during SS and SW, while it was close to or greater than zero (0.02 s < T^F^ < 0.94 s) during IDS and TDS, except for T_AP_ during OG walking at FAST and SS speeds. Between OG and RE walking, unlike MoS_AP_ outcomes, T_AP_ values were relatively similar.

## Discussion

Previously, walking performance involving RE-assisted gait has included clinical measures, functional measures, and gait analysis studies focused primarily on spatiotemporal and lower limb kinematic outcomes during quadrupedal gait using inverse dynamic techniques (Sylos-Labini et al., 2014; Ramanujam et al., 2017, 2018, 2019a,b; Husain et al., 2018; Forrest et al., 2019; Wang et al., 2019). Moreover, our group has not only published articles on upper and lower extremity kinematics but also studied the posture and balance of individuals (both SCI and AB controls) during RE-assisted gait with forearm crutches by examining their instantaneous CoM excursions (whole body, trunk, and lower extremity) in relation to the BoS (Ramanujam et al., 2019a,b). For this manuscript, we have combined all of these parameters to determine the instantaneous MoS for investigating stability control using different RE devices. To date, there is limited research to evaluate balance control using different powered RE devices. In this study, human–machine interaction for computed dynamic margins of stability were quantified for walking performance during RE-assisted walking compared to independent OG walking for healthy adults.

### MoS Outcomes vs. Speed During OG and RE Walking

MoS outcomes were negatively correlated (*p* < 0.05, Table 3) to walking speed during OG walking. Therefore, with an increase in walking speed, instability increased in both AP and ML directions. By comparison, 80% (8/10 correlations) of RE conditions showed weak (|*R*| < 0.37) correlations for either direction. Therefore, control mechanisms for stability using REs were not related to changes in walking speed. The exceptions were the Indego® (*p* < 0.05) medial–laterally and EksoGT™ (*Max-Assist* mode; *p* < 0.05) in the AP direction.

For many REs, device parameters such as the assist modes or motor assistance levels determined the human–machine interactions affecting walking speed. The greater stance time required to meet the forward and lateral targets for step initiation translated to slower walking speeds (Sylos-Labini et al., 2014; Ramanujam et al., 2017), whereas faster speed required rapid and spatially extensive weight shifts (predominantly by the trunk and pelvis) onto the lead limb for a quicker step initiation with the trail limb during stance to increase CoM velocity and negatively affect the margins of stability. This is especially true while walking with the EksoGT™ (assist modes) and ReWalk™ since they operate on the principle of lateral and anterior weight shifts, respectively, on to the lead limb, to initiate the next step. This delay in weight acceptance can affect overall walking speed. By comparison, for OG walking, dynamic stability can be controlled by lower extremity foot placement, especially lateral foot placement (Hof, 2008). These progressive step changes in foot placement are essential to walking stability in the prevention of disturbances such as slips and falls (Kennaway, 1970; Bennett and Murphy, 1977; Bateni and Maki, 2005; Saunders et al., 2013).

Crutches for quadrupedal gait increase the BoS to improve balance (Bateni and Maki, 2005; Saunders et al., 2013). However, assistive devices have been associated with an increased risk of falling and injuries during the expected or unexpected transition (crutch lift or slippage) from quadrupedal to bipedal gait during non-exoskeleton gait (Bateni and Maki, 2005). Lifting or slippage of the assistive device is similar to lifting the foot, causing the CoM to fall toward the unsupported side during unassisted gait, creating a state of imbalance where the CoM lies outside the limits of BoS (Bateni and Maki, 2005). During RE walking (especially, EksoGT™), the devices' limitation toward choosing the desired lateral foot placement puts more emphasis on crutch location outside the leading limb to provide a stable BoS, resulting in reduced ML control of stability. The location of this crutch may also be influenced by the different surfaces (e.g., carpet, pavement) (Wang et al., 2019).

### Device Operation and its Effect on Stability

In addition to moderate-to-strong relationships between MoS outcomes and walking speed, for OG gait, MoS_AP_ was significantly positive and moderately correlated (*p* < 0.05) to MoS_ML_. Therefore, during OG gait, the mechanisms for controlling dynamic stability were multi-planar and changed based on gait speed. For RE devices, there were very weak and non-significant relationships between MoS_AP_ and MoS_ML_ except for ReWalk™ and EksoGT™ (*2Free*), which exhibited positive and negative relationships for MoS_AP_ and MoS_ML_, respectively. Therefore, gains in stability were bi-planar only in the ReWalk™.

The inherent differences in the design and control mechanisms of these devices altered the way users maintain balance and control their dynamic stability. As found earlier, the control of MoS was not necessarily related to speed during RE walking. The changes in MoS during RE walking were governed more by the positional aspect of the CoM due to the necessary ML and/or AP weight shifts for step initiation rather than its velocity component. For instance, the EksoGT™ operates on the principle of lateral weight shift onto one foot to complete the stepping on the contralateral foot combined with moving the contralateral crutch forward, while the ReWalk™ uses a “tilt” action that initiates a step by tilting the trunk anteriorly and moving both crutches forward simultaneously. This increase in trunk lean angle at heel-strike and greater AP excursion of CoM while walking with the ReWalk™, as previously reported in our earlier study (Ramanujam et al., 2019a), translated into a more anterior location of the CoM, resulting in lower stability values (for MoS^F^) in the AP direction compared to EksoGT™ (*Max-Assist* and *Fixed-Assist*). Similar is the case with AP stability in the Indego® that uses postural cues to trigger all transitions (e.g., to walk forward, the user just leans forward) and EksoGT™ (*2Free* mode) where the user is free to move in any direction while stepping and not restricted to ML weight shifts.

It should also be noted that each standard deviation associated with the mean MoS (ML or AP) for Fixed-Assist modes (Table 3) is low, indicating that the difference in levels of assistance in the Fixed-Assist mode (60, 35, 0%) had a minimal effect on MoS values.

### Effect of Crutch Placement on Stride-to-Stride Control

Stride-to-stride variability in MoS outcomes is indicative of the control of stability during consecutive steps. The dispersion of points on the first-return plots determines step-to-step adaptations during gait. For OG walking, as expected, the stride-to-stride variability was significantly low except for FAST speeds in the AP direction compared to RE devices. One of the major determinants of faster walking speed is increased step and stride lengths. The variability in terms of anterior foot placements across strides and individuals, to increase step lengths and achieve faster speeds, translated into higher RMSD_AP_ values. During RE walking, the foot placements are, for the most part, governed and limited by the device settings and hence quite similar from one step to another. However, the placement of bilateral crutches varies stride to stride, across individuals and devices/modes. The precise location of crutch placement is based on individual preferences. Therefore, variability in crutch placement translates into higher dispersion of stride-to-stride stability measures and, hence, reduced control of stability especially during the transition to a bipedal gait.

For RE walking, RMSD_ML_ values were found to be significantly greater (*p* < 0.05) with EksoGT™ for the *Max-Assist* and *Fixed-Assist* modes, while RMSD_AP_ values were significantly greater with the Indego® and ReWalk™. During *Max-Assist* (EksoGT™), individuals tend to load onto their leading limb to achieve the required lateral weight shift for step initiation with the trailing limb. Hof (2008) found in their study that stability might be maintained or controlled by the lateral foot placement during walking. However, during RE walking (especially EksoGT™), the extent of lateral foot placement is restricted by the device design and setting. Consequently, the crutch is more lateral to the leading limb to provide a stable BoS, resulting in higher RMSD_ML_ values and reduced ML control of stability. This is especially relevant to the *Fixed-Assist* mode. Conversely, with the ReWalk™ and Indego®, the emphasis is on forward trunk lean for step initiation and hence a more anterior location of crutches. This results in greater AP stride-to-stride variability in MoS and a reduced AP control of stability.

### Control of Stability During a Gait Cycle

As noted earlier, for quadrupedal RE gait, the majority of postural adaptations and weight transfer within each device occur during the phases of SS just prior to step initiation. As a result, all stability outcome measures are lower during these phases.

Dynamic margins of stability in the ML direction were found to be stable during the double support phases of OG walking at all speeds as well as RE walking across the tested devices. Anterior–posteriorly, all three devices were most stable for both the crutch (MoS^C^) and foot (MoS^F^) margins of stability during double support. However, instability was observed during SS (MoS^F^ only). In the EksoGT™, although the *Max-Assist* and *Fixed-Assist* modes necessitate users to shift their weight laterally to complete the stepping motion, the *2Free* mode provides more freedom to translate or step anteriorly. This is supported by lower values of MoS_AP_ in the *2Free* mode of EksoGT™ walking during SS. Similar observations were seen while walking in the ReWalk™ and Indego®, which requires users to lean forward with their trunk for step initiation.

Since the analyses for MoS outcomes for RE were not related to speed and there were large device differences for walking speeds compared to OG walking, MoS outcomes were normalized relative to speed for each phase of a gait cycle, defined as the temporal stability margin (T). T^F^ in the AP direction during RE walking was similar to OG walking at SLOW and SS speeds. Therefore, the RE gait stability based on MoS parameters normalized to speed was like OG gait. Therefore, despite the assistance and postural support offered by the RE, it did not necessarily alter the AP stability measures compared to OG walking. Of note, while walking with EksoGT™ in the *2Free* mode, the temporal AP stability with respect to feet (T^F^) as well as crutches (T^C^) was much lower compared to other RE devices and modes during SS.

### Significance of MoS Referenced to Feet

Since RE walking occurred exclusively with the use of bilateral forearm crutches and at relatively low speeds (<0.6 m/s), the mean MoS outcomes (MoS^C^) were significantly greater than those experienced during independent OG walking across all speeds (>0.8 m/s). The wider BoS provided by the crutches compared to just the feet (OG walking) increased the overall dynamic stability for a quadrupedal gait for all RE devices tested. However, although the margins of stability were high with crutch usage, it is still important to examine and evaluate the postural orientation of the whole-body CoM relative to feet (bipedal gait). In the event of crutch slippage caused by a variety of reasons, the feet will act as a bipedal BoS for individuals during RE walking. To further examine this, MoS outcomes were also computed relative to feet as the BoS (MoS^F^), which were found to be considerably lower compared to those calculated relative to crutches (MoS^C^).

Bilateral stability outcomes relative to feet, both MoS^F^ and T^F^ (Figure 4), are considerably lower throughout the gait cycle compared to those calculated relative to crutches (MoS^C^ and T^C^). During the SS phases of RE walking, majority of postural adjustments and ML weight transfers occur to initiate stepping with the trail limb. Using the crutch during RE walking is not only precautionary to provide support, but individuals tend to lean on the crutches during these phases, especially medial–laterally (for EksoGT™) and anterior–posteriorly (for, ReWalk™ and Indego®), in order to propel themselves forward. As a result, all the stability outcome measures are lower during these phases.

Assistive devices can inhibit balance during gait to increase fall risk (Kennaway, 1970; Phonthee et al., 2013). Crutch slippage due to the material used or different walking surfaces (e.g., wet pavements, snow, ice, etc.) can lead to injuries (Kennaway, 1970; Bennett and Murphy, 1977). MoS data and analyses indicate that the reliance on crutches during RE walking moves the CoM laterally and in some cases beyond the BoS defined by the feet, thereby increasing postural instability and fall risk if the crutch were to slip.

### Limitations

While the results provided significant insight into human–robotic interaction for stability during RE gait and directly addressed the hypothesis to show significant differences in the MoS measures during RE-assisted walking based on device, compared to independent OG walking for all speeds, these results are preliminary. A greater number of trials for all conditions and number of training sessions per individual are needed to further analyze stability. The mechanical measures determined the dynamic MoS without consideration given to the difference in neuromuscular strategies, for recovery of gait and balance. Research in these areas is required for community and rehabilitation devices.

### Conclusion and Future Works

For healthy adults, stability outcomes alone and their relationship to walking speed during RE walking compared to independent OG walking were significantly different. Due to exoskeleton design, margins of stability or control mechanisms for stability during RE walking were not related to walking speed. Despite the dissimilarities in the design and operation of these RE devices, the dynamic margins of stability for these individuals were found to be lower during SS, especially in the ML direction across all devices. Further, the reliance on crutches and their spatial location during RE walking pushed the CoM, during SS, beyond the lateral boundary of the lead foot, thereby placing the individuals at risk for falls if crutch slippage was to occur, especially relevant when individuals cannot recover from an unbalanced posture. Consideration of crutch placement is therefore relevant to stride-to-stride postural control and margins of stability within the limits of bipedal BoS for dynamic stability. Understanding the interactions between humans, RE devices, and assistive devices (if used) combined with training adaptions is relevant to the advancements in the field of exoskeleton technology, both in research and in the clinic. Future research will include a more comprehensive analysis of the different assist modes within each exoskeleton and the possible use of the Monte Carlo statistical technique to further evaluate the associated outcome measures.

## Data Availability Statement

The raw data supporting the conclusions of this article will be made available by the authors, without undue reservation.

## Ethics Statement

The studies involving human participants were reviewed and approved by Kessler Foundation and James J. Peters VA Medical Center's Institutional Review Boards. The patients/participants provided their written informed consent to participate in this study.

## Author Contributions

All authors listed have made a substantial, direct and intellectual contribution to the work, and approved it for publication.

## Conflict of Interest

The authors declare that the research was conducted in the absence of any commercial or financial relationships that could be construed as a potential conflict of interest.
